# Risk of Thrombosis in Adult Philadelphia-Positive ALL Treated with an Asparaginase-Free ALL Regimen

**DOI:** 10.3390/curroncol28010016

**Published:** 2020-12-22

**Authors:** Ruiqi Chen, Xing Liu, Arjun D. Law, Solaf Kanfar, Dawn Maze, Steven M. Chan, Vikas Gupta, Karen W. Yee, Mark D. Minden, Aaron D. Schimmer, Andre C. Schuh, Caroline J. McNamara, Tracy Murphy, Anna Xu, Umberto Falcone, Jack Seki, Hassan Sibai

**Affiliations:** 1 Princess Margaret Cancer Centre, Department of Medical Oncology and Hematology, University Health Network, Toronto, ON M5G 2C1, Canada; ruiqi.chen@mail.utoronto.ca (R.C.); arjun.law@uhn.ca (A.D.L.); solaf.kanfar@kfsh.med.sa (S.K.); dawn.maze@uhn.ca (D.M.); steven.chan@uhn.ca (S.M.C.); vikas.gupta@uhn.ca (V.G.); karen.yee@uhn.ca (K.W.Y.); mark.minden@uhn.ca (M.D.M.); aaron.schimmer@uhn.ca (A.D.S.); andre.schuh@uhn.ca (A.C.S.); caroline.mcnamara@uhn.ca (C.J.M.); tracy.murphy@uhn.ca (T.M.); jack.seki@uhn.ca (J.S.); 2 Leslie Dan Faculty of Pharmacy, University of Toronto, Toronto, ON M5S 3M2, Canada; lindalinda.liu@mail.utoronto.ca (X.L.); anna.xu@unityhealth.to (A.X.); 3 Department of Pharmacy, Princess Margaret Cancer Centre, University Health Network, Toronto, ON M5G 2M9, Canada; 4 Department of Haematology, Istituto Nazionale Tumori IRCCS Fondazione Pascale, 80131 Naples, Italy; umberto80@libero.it

**Keywords:** venous thromboembolism, acute lymphoblastic leukemia, philadelphia-positive acute leukemia, cancer-related thrombosis, treatment-related thrombosis

## Abstract

Background: venous thromboembolism (VTE) is a well-known complication in adults with acute lymphoblastic leukemia (ALL), especially in patients treated with asparaginase (ASNase)-including regiments. However, VTE risk in adult Philadelphia-positive ALL (Ph+ve ALL) patients treated with non-hyperCVAD chemotherapy is unclear. In this study, we examined VTE incidence in adult Ph+ve ALL patients treated with imatinib plus a pediatric-inspired asparaginase (ASNase)-free regimen modified from the Dana Farber Cancer Institute (DFCI) ALL protocol. Methods: a single centre retrospective review of Ph+ve ALL patients treated at Princess Margaret Cancer Center (PMCC) from 2008–2019 with imatinib plus modified DFCI protocol was conducted. Results: of the 123 patients included, 30 (24.3%) had at least 1 radiology confirmed VTE event from diagnosis to the end of maintenance therapy. 86.7% (26/30) of the VTE events occurred during active treatment. Of all VTE events, the majority (53.3%) were DVT and/or PE while another significant portion were catheter-related (40.0%). Major bleeding was observed in 1 patient on VTE treatment with low molecular weight heparin (LMWH). Conclusion: a high VTE incidence (24.3%) was observed in adults Ph+ve ALL patients treated with imatinib plus an ASNase-free modified DFCI pediatric ALL protocol, suggesting prophylactic anticoagulation should be considered for all adult Ph+ve ALL patients including those treated with ASNase-free regimens.

## 1. Introduction

Venous thromboembolism (VTE) is a well-known complication in oncology patients, either as a presenting symptom or as a complication of treatment [1]. Active neoplasms create a hypercoagulable state through several mechanisms. Neoplasms release increased tumour procoagulants, activate platelets, and increase endothelial tissue factor expression. Simultaneously, physiological anticoagulants and fibrinolytic activity are reduced [2]. Hematological malignancies have an elevated VTE risk that is comparable to high-risk solid tumours including brain, pancreatic, and ovarian cancer [3].

Acute lymphoblastic leukemia (ALL) are classified in two main subcategories, Philadelphia-negative (Ph-ve) and Philadelphia-positive (Ph+ve) ALL, as chemotherapeutic approaches are different [4]. Ph+ve ALL is characterized by a t(9;22) translocation and is found in approximately 20% of ALL patients [5]. Previous reports showed that VTE incidence in ALL patients ranges from 10.3% to 40.8% [6,7,8,9]. Several risk factors including age, sex, Philadelphia chromosome status, and asparaginase (ASNase) treatment were associated with VTE incidence in ALL patients [7]. ASNase is a well-known risk factor as it can lead to a significantly increased risk for VTE development, in large via mechanisms through the inhibition of hepatic protein synthesis including antithrombin III and protein C/S [10,11]. As a result, the prevention of VTE complications in patients receiving ASNase-based chemotherapy has remained in focus [12]. Several studies show that anticoagulation prophylaxis may be recommended in Ph-ve ALL patients as most receive ASNase at some point [9,13]. However, this is not a standard recommendation in adult Ph+ve ALL, which is typically treated with chemotherapy in combination with a tyrosine kinase inhibitor (TKI) [14,15,16,17,18,19]. Previously, the MD Anderson Cancer Centre reported an overall VTE incidence of 29% (*n* = 14/49) in Ph+ve ALL patients treated with the Hyper-CVAD protocol and established that Philadelphia chromosome positivity alone was a risk factor for VTE compared to Ph-ve ALL (OR: 2.7, 95% CI: 1.21–6.21) [7].

The incidence of VTE in adult Ph+ve ALL patients treated other protocols requires further research. Our centre uses imatinib plus a pediatric-inspired regimen modified from the Dana Farber Cancer Institute (DFCI) protocol [20]. Of note, the current modified DFCI protocol for Ph+ve ALL does not contain ASNase, which has been removed from our practice in 2008 due to unacceptable hepatic toxicity. Herein, we describe our experience with imatinib in combination with an ASNase-free pediatric inspired protocol in adults with Ph+ve ALL and examine the patterns of VTE in this population.

## 2. Methods

This study entails a single centre retrospective chart review of Ph+ve ALL patients treated at Princess Margaret Cancer Center (PMCC) from 2008–2019 with an imatinib-containing DFCI protocol-based regimen that omitted ASNase. Patients were identified from the hospital leukemia database. Patient electronic medical records and hospital pharmacy records were reviewed. Institutional ethics review board approval was obtained (Approval by The University Health Network Research Ethics Board, code 15-5077.4, 25 April 2016).

**Figure 1:**
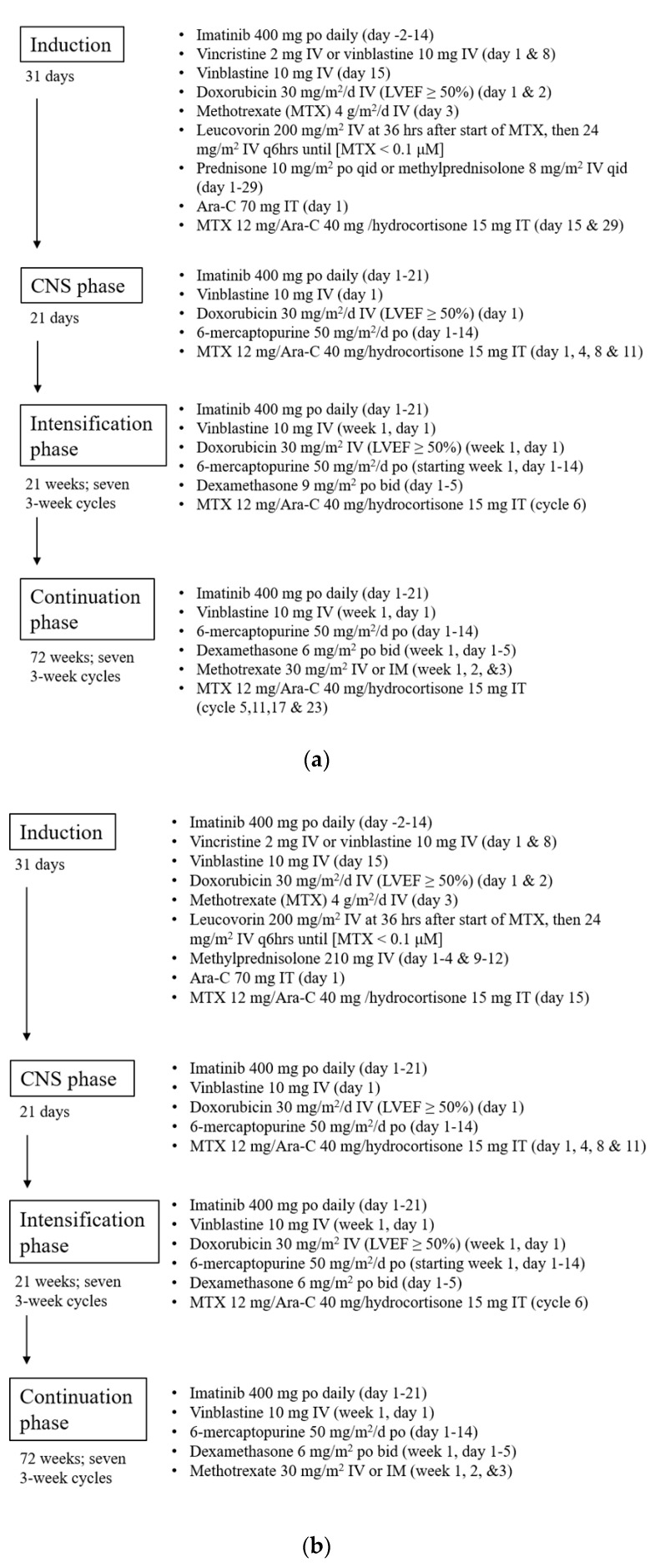
(**a**). Modified Dana Farber Cancer Institute (DFCI) protocol for adult Ph+ve acute lymphoblastic leukemia (ALL) patients with age under 60. (**b**). Modified DFCI protocol for adult Ph+ve ALL patients with age above 60.

The treatment regimen under the modified DFCI protocol is summarized in Figure 1a, while patients above 60 years old received a reduced intensity protocol described in Figure 1b. Of note, allogeneic bone marrow transplant (BMT) was traditionally offered to all adult Ph+ve patients after receiving induction chemotherapy, but the practice has been evolving as continuation with chemotherapy + TKI in patients who showed >3 log reduction after induction has gained popularity in recent years [21]. Patients were included in this study if ≥18 years old, with a confirmed diagnosis of Ph+ve ALL, underwent imatinib plus the ASNase-free modified DFCI protocol, and achieved complete morphological remission after induction chemotherapy. Patients who received VTE prophylaxis were excluded from the analysis. Patients were followed until they completed the modified DFCI protocol, underwent BMT, relapsed, stopped DFCI regimen early due to intolerance, died during treatment, or were lost to follow up.

The primary objective for this study was to characterize the incidence of VTE in adults with Ph+ve ALL during the entire ASNase-free DFCI treatment course. All VTE events were radiologically proven, including PE, DVT, line-related thrombosis, or others. Line-related thrombosis was categorized as PICC-line related and central venous catheter (CVC) related. The incidence of VTE was calculated as a fraction of all patients that underwent the modified DFCI treatment rather than those who actually completed each phase. The former method was chosen as a conservative measurement to preclude overestimation. Co-variants of age, sex, weight, BMI, follow-up time, Padua score, white blood cell count, and platelet count were also collected.

Descriptive statistics are used to summarize baseline patient demographics, disease characteristics, and laboratory data. Categorical variables such as sex, VTE proportions, and VTE sites are summarized with counts and percentages. Continuous variables such as age at diagnosis and weight are expressed as means ± standard deviation or medians with ranges. Categorical variables were analyzed using chi-square or Fisher exact tests when appropriate. Continuous variables for the 2 groups were compared using a two-tailed student’s *t*-test for parametric tests and Wilcoxon signed-rank test for nonparametric test. The cumulative incidence of VTE was calculated from the time of diagnosis to the end of modified DFCI treatment, including at the time of allogeneic bone marrow transplantation. All *p*-values were two-sided, and *p* < 0.05 was considered to indicate a significantly different result.

## 3. Results

Overall, 123 patients were included in our analysis with baseline characteristics summarized in Table 1. The mean age at the time of diagnosis was 49.9 years (range 17.4–80.9 years), with 45.5% being females. The average weight of the whole cohort was 74.7 kg (41.7–127 kg) and the average BMI was 26.4 kg/m^2^ (18.9–52.2 kg/m^2^). All patients received imatinib as the first-line TKI and completed modified DFCI induction. Of the 123 patients, 90 (73.2%) received the standard protocol (Figure 1a) while 33 (26.8%) received reduced intensity DFCI due to age >60 (Figure 1b). Notably, among the 123 patients, 106 (86.2%) completed induction, 82 (66.7%) completed intensification, and 48 (39.0%) completed the full treatment course. Among the patients who did not complete the DFCI protocol, 44 proceeded to bone marrow transplant, 14 passed away, 13 had relapsed, 4 stopped due to toxicity, and 1 was lost in follow up.

Overall, 30 (24.3%) patients had at least 1 VTE events from diagnosis to the end of treatment. The mean age was older in patients who developed VTE versus those who did not (*p* = 0.010). There was no statistically significant difference between patients who developed VTE in comparison to patients who did not develop VTE in terms of sex, weight, BMI, WBC count, or blast percentage (Table 1). The mean Padua score for these patients was 5.4 with a range of 3–10.

**Figure 2:**
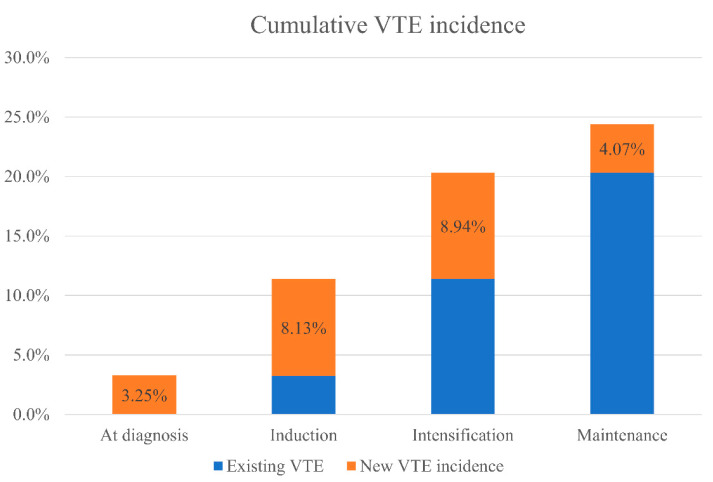
Overall temporal distribution of the 30 VTE events from diagnosis to the end of maintenance with respect to the whole cohort of 123 patients. The incidence was 3.25% (4 out of 123) at diagnosis, 8.13% (10 out of 123) during induction, 8.94% (11 out of 123) during intensification, and 4.07% (5 out of 123) during maintenance.

**Figure 3:**
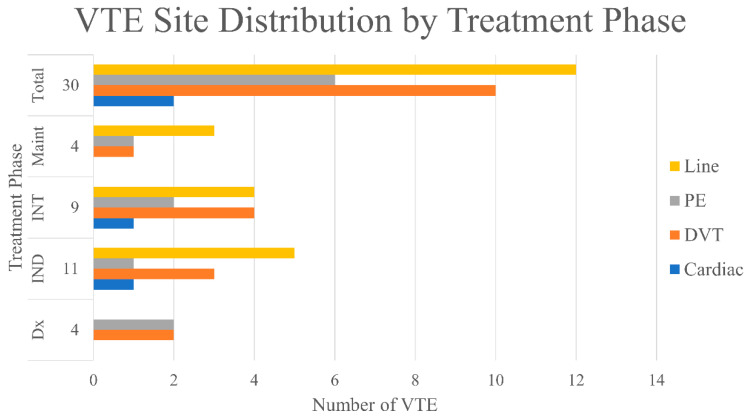
VTE site distribution throughout treatment phase. Dx = diagnosis; IND = induction; INT = intensification; Maint = maintenance.

The majority of the VTE events occurred during active modified DFCI treatment plus imatinib, with 10 (8.1%), 11 (8.9%), and 5 (4.1%) occurring during induction, intensification, and maintenance, respectively (Figure 2). Only 4 patients (3.1%) had VTE at the time of leukemia diagnosis. Of all 30 patients developed VTE, 16 (53.3%) were DVT and/or PE, 12 (40.0%) were PICC/CVC-related, and 2 (6.7%) involved a cardiac thrombus (Figure 3). For line-related thrombosis, 3 events were noted among 79 patients with CVC (~4% incidence), whereas 9 events were found from 44 patients on PICC-line (~20% incidence). A second thrombotic event during the modified DFCI treatment happened in 2 patients while on low molecular weight heparin (LMWH) treatment. With regard to bleeding risk, of the 10 patients who developed VTE during induction, 9 had days with platelet level of less than 50 bil/L (Table 2). In contrast, of the 11 patients who had VTE during intensification, only 2 had days with platelet level of less than 50 bil/L. When patients developed VTE during treatment, chemotherapy was temporarily held for 2 weeks and full-dose tinzaparin or enoxaparin was initiated. Two patients had experienced a recurrent VTE episode within a year while on LMWH treatment.

In terms of safety, only major bleeding events were collected in accordance to the guideline from International Society of Thrombosis and Haemostasis (ISTH) [22]. Major bleeding following anticoagulation was rare, which happened in 1 of the 30 patients who developed VTE throughout the treatment course followed by LMWH treatment. This patient developed a right atrial clot during the induction phase while in the ICU, and experienced a major bleeding into the psoas muscle while on LMWH plus platelet transfusion treatment. In this patient, LMWH was withheld for at least 1 week, and during this time no breakthrough VTE event occurred. No major bleeding events were observed in any of the other patients, including those who received therapeutic doses of LMWH after being diagnosed with VTE.

## 4. Discussion

The incidence of thrombosis in Ph+ve ALL is not well understood. In this study, we describe the largest adult Ph+ve ALL patient cohort published to date to characterize the thrombosis risk in this group. We have demonstrated that Ph+ve ALL patients receiving an ASNase-free protocol have a high risk of VTE development (30/123, 24.3%) from diagnosis to the end of treatment, with the majority of VTE events happening during induction and intensification phases (Figure 2). Although the incidence of VTE was slightly lower than the previously published VTE incidence (29%) in adult Ph+ve ALL patients receiving Hyper-CVAD [23], the results from our cohort suggest that the risk of thrombosis may have been underestimated in patients receiving ASNase-free protocols.

With regard to the site of VTE development, half of VTE events were DVT and/or PE (Figure 3). Catheter-related thrombosis also accounted for 40.0% of total VTE events and 9.8% of the total patient cohort, but this incidence was similar to previously reported central line-related thrombosis in cancer patients [24]. These results suggest that the high VTE incidence in Ph+ve ALL patients is multifactorial rather than related to leukemia alone [25,26]. In this study, age was a statistically significant cofactor associated with VTE (*p* = 0.010). This may be an independent predictor for VTE risk, but also may be a surrogate factor for underlying reasons, such as potentially more physical inactivity, artherosclerosis, and endothelial damages. Other factors may include chemotherapy other than ASNase (i.e., doxorubicin [27]), high dose corticosteroids [28], and PICC line use [24]. In fact, PICC-line related thrombosis happed in approximately 20% of patients while CVC-related thrombosis was only observed in 4% of patients. The decision of choosing CVC vs. PICC lines currently mainly depends on availability and patient preferences. This finding is another reminder that CVC may be preferred over PICC line from a thrombosis standpoint. Interestingly, in our practice, we only diagnosed line-related thrombosis in symptomatic patients with doppler ultrasound or incidental finding by CT scan. The full extent of line-related thrombosis in this group of patients would require a screening process that may warrant further studies. Of note, imatinib and dasatinib are not known to be pro-thrombotic [29,30,31]. We did not include patients who received ponatinib since we do not use it in the first-line setting, as well as its known thrombogenic effects.

The high thrombosis risk in Ph+ve ALL patients treated with the ASNase-free DFCI raises the question of whether anticoagulation prophylaxis is warranted. Previous studies of anticoagulation prophylaxis in Ph-ve ALL patients treated with ASNase-containing DFCI protocol showed a significant reduction of VTE with daily low molecular weight heparin (LMWH) injections [9,13,32]. A major concern for anticoagulation prophylaxis is the risk of bleeding as ALL patients tend to have concurrent thrombocytopenia and hypercoagulability. One incidence of potentially LMWH-related major bleeding was noted in this study, suggesting that albeit low, there is a chance for major bleeding in patients treated with LMWH. Therefore, there is a need to carefully select patients with respect to patient factors, phase of treatment, choice of anticoagulant, dosing strategies in the presence of thrombocytopenia, and the impact of central venous catheters which warrant further studies.

Traditionally, LMWH has been used for anticoagulation prophylaxis, but its need for daily injections and continued VTE breakthrough from LMWH prophylaxis had led to considerations of direct oral anticoagulants (DOACs) [33]. Since recent studies showed the effectiveness of DOACs as prophylaxis agents as well as treatment for VTE in adults with solid tumours [34,35,36], further studies are needed to confirm the efficacy and safety of both LMWH and DOACs for VTE prophylaxis in Ph+ve ALL patients.

Our study is limited for its retrospective and descriptive nature. Therefore, several important factors such as comorbidities and minor bleeding events cannot be determined accurately, hindering the identification of potential predictors and safety profile characterizations. In addition, the retrospective nature of the study also did not allow us to fully elucidate asymptomatic thrombosis events such as calf vein thrombosis and incidental PE. Importantly, incidental PE may be a factor that contributes to cancer-related mortality [37,38] that were not captured in this study. Future prospective verifications will be needed to provide further insights into VTE risk and potential management options.

## 5. Conclusions

VTE was observed in 24.3% of adults with Ph+ve ALL patients treated with imatinib plus an ASNase-free modified DFCI pediatric ALL protocol. The majority of VTE events occurred during active treatment phases such as induction and intensification. DVT/PE consisted the majority of the thrombosis events while line-related thrombosis was also significant. The high observed VTE incidence suggests that future anticoagulation prophylaxis studies should include Ph+ve ALL instead of just focusing on ASNase-based treatment for Ph-ve ALL patients, especially if additional thromboembolic risk factors are present.

## Figures and Tables

**Table 1:** Baseline Patient Characteristics.

Variables	Whole Cohort (*n* = 123) Mean (Range)	Patients Who Developed VTE (*n* = 30) Mean (Range)	Patients Who Did Not Develop VTE (*n* = 95) Mean (Range)	*p* Value
Age at diagnosis (range)	49.9 (17.4–80.9)	55.2 (24.2–80.9)	48.2 (17.4–80.0)	0.01
Sex, female (%)	56 (45.5%)	16 (53.3%)	40 (43.0%)	0.32
Weight in kg (range)	74.7 (41.7–127)	73.1 (41.7–110.8)	74.7 (47.0–127.0)	0.48
BMI in kg/m^2^ (range)	26.1 (18.9–52.2)	26.3 (18.9–38.3)	26.4 (17.7–52.2)	0.56
WBC in bil/L (range)	45.6 (0–460)	41.9 (1.4–413)	46.8 (0–460)	0.84
Blast in % (range)	72.8 (0–98)	74.9 (0–98)	72.1 (0.8–97)	0.80

**Table 2:** Characteristics of patients with venous thromboembolism (VTE).

Variables	Sub-Categories	Patients with VTE *n* = 30 *n* (%)
Timing of VTE	At diagnosis	4 (13.3)
	Induction	10 (33.3)
	Intensification	11 (36.7)
	Maintenance	5 (16.7)
Location of VTE	DVT	10 (33.3)
	PE	6 (20.0)
	Cardiac	2 (6.7)
	PICC/CVC-related	12 (40.0)
plt < 50 bil/L	Among 10 VTE pts during induction	9 (9 in 10, 90.0%)
	Among 11 VTE pts during intensification	2 (2 in 11, 18.2%)
Padua score (mean, range)		5.4, 3–10
